# Magnetohydrodynamic Simulations of Hypersonic Flow over a Cylinder Using Axial- and Transverse-Oriented Magnetic Dipoles

**DOI:** 10.1155/2013/438381

**Published:** 2013-11-06

**Authors:** Andrew N. Guarendi, Abhilash J. Chandy

**Affiliations:** Department of Mechanical Engineering, The University of Akron, Akron, OH 44325-3903, USA

## Abstract

Numerical simulations of magnetohydrodynamic (MHD) hypersonic flow over a cylinder are presented for axial- and transverse-oriented dipoles with different strengths. ANSYS CFX is used to carry out calculations for steady, laminar flows at a Mach number of 6.1, with a model for electrical conductivity as a function of temperature and pressure. The low magnetic Reynolds number (≪1) calculated based on the velocity and length scales in this problem justifies the quasistatic approximation, which assumes negligible effect of velocity on magnetic fields. Therefore, the governing equations employed in the simulations are the compressible Navier-Stokes and the energy equations with MHD-related source terms such as Lorentz force and Joule dissipation. The results demonstrate the ability of the magnetic field to affect the flowfield around the cylinder, which results in an increase in shock stand-off distance and reduction in overall temperature. Also, it is observed that there is a noticeable decrease in drag with the addition of the magnetic field.

## 1. Introduction

 Space agencies including NASA have been currently engaged in research to develop low-cost alternatives for access-to-space and novel concepts for high Mach number or hypersonic propulsion. Hypersonic vehicles require essential improvements in order to ensure economic viability and to fulfill mission and safety constraints. For the last two decades various methods have been investigated to control hypersonic air flows specifically with regard to (a) lowering heat transfer to high-speed flying bodies, (b) inlet flow optimization in scramjets, (c) wave drag and wave turbulence cancellation, and (d) braking for atmospheric reentry. More recently there has been an intense interest in using MHD techniques to control the flow, and their potential applications are aimed at improving the performance during off-design conditions [1]. Besides the use of plasmas in space thrusters, a growing interest is evident in plasma-based aerodynamics, including flow manipulation through MHD forces, and drag reduction [2–6]. The size and complexity of this problem have led to growing importance of numerical methods for design and optimization, especially due to the difficulties of reproducing the MHD interactions around a hypersonic vehicle in laboratory test facilities. Recent developments in Computational Fluid Dynamics (CFD) such as increased robustness of CFD codes, lower computational costs, and improvements in hardware as well as grid generation and more user-friendly postprocessing tools can reduce dramatically the design and development time required for new vehicles.

Magnetohydrodynamics (MHD) concerns the flows of electrically conducting fluids in the presence of a magnetic field. These flows obey the coupled Navier-Stokes (NS) and Maxwell equations and are accompanied by the occurrence of induced electric currents within the fluid, which leads to Joule dissipation [7]. The suppression of motion of these fluids by a static magnetic field is a subject of increasing interest in many technologies [7, 8] including aerospace. In such MHD applications, the magnetic Reynolds number Re_*m*_ = *UL*/*η* ≪ 1 (*U* and *L* are velocity and length scales, resp., and *η* is the magnetic diffusivity). At low Re_*m*_, one can invoke the quasistatic approximation in MHD, where the influence of the velocity field on MHD is assumed to be negligible.

Some of the earliest works included [9–12]. Later, Coakley and Porter [13] developed the first CFD simulations of an MHD blunt body problem, though with simplifications in order to reduce computational effort. In the late 1990s and early 2000s, interest in MHD effects began to spring anew, as materials research began catching up and further demand for increased capability in space by both national and commercial entities emerged [14–17]. Furthermore, the revelation of a Soviet hypersonic plane—the AJAX project—demonstrated for the first time a potential real-world application of hypersonic MHD flow control. This work on MHD hypersonic flow was continued by many researchers [18–21], who conducted both physical experiments and computational methods for hypersonic MHD flow over blunt bodies. More recently, Grigoriadis et al. [22] computed hypersonic MHD flow past a cylinder using the immersed boundary method. Bisek et al. [23] investigated the effects of a magnetic field on hypersonic flow using variable electrical conductivity models and an argon gas medium in considering its application as a heat shield for reentry vehicles. Over the last few years, development of hypersonic MHD applications has begun to cluster into flow control, hypersonic inlets, and power generation [24–26]. For instance, depending on the orientation and magnitude of the magnetic field, drag can be either increased or decreased around a cylinder, [22, 27, 28] and the location of the shock wave can be altered, potentially dissipated, or even eliminated [29].

In this era of the retirement of the Space Shuttle, the growth of space programs around the world, and the emergence of commercial entities such as SpaceX (http://www.spacex.com/), MHD looks to play an ever increasing role in the future of aerospace. This paper examines hypersonic laminar flow of partially ionized air over a cylinder with an applied magnetic field in the form of a dipole oriented in the axial and transverse directions. Particular focus is given to the resulting change in flow patterns, heat transfer, and drag forces.

## 2. Formulation

The governing equations solved in this study are the compressible Navier-Stokes equations along with the magnetic effects in the form of the Lorentz force in the momentum equation and the Joule dissipation term in the energy equation. With the steady-state assumption, the equations are given by

(1) $$\begin{array}{c} \nabla \cdot \left(\rho U\right)=0,\\ \nabla \cdot \left(\rho U⊗U\right)=-\nabla p+\nabla \cdot \tau +F_{\text{Lor}},\\ \nabla \cdot \left(\rho Uh_{\text{tot}}\right)=\nabla \cdot \left(\lambda \nabla T\right)+\nabla \cdot \left(U+\tau \;\right)+J_{H}, \end{array}$$

where the stress tensor **τ** is related to the strain rate by

(2) $$\begin{array}{c} \tau =\mu \left(\nabla U+{\left(\nabla U\right)}^{T}-\frac{2}{3}\delta \nabla \cdot U\right).\; \end{array}$$

## 3. Problem Description

The problem studied consists of the flow of partially ionized air over a cylinder at hypersonic speeds. The flow is assumed to be laminar and steady with a reference pressure of 85 Pa. The medium is treated as an ideal gas, with no chemical equilibrium or real gas effects. The inlet velocity is 2088.6 m/s at a temperature of 291.44 K and a Mach number of 6.1. The viscosity was adjusted so as to have the calculated Reynolds number for this flow as

(3) Re=ρULμ≈234.

These far-field temperature and pressure values are those of air at an altitude of 50,000 m [30].

A constant magnetic field is applied and is modeled as a dipole centered at the center of the cylinder. However, the dipole is essentially treated as a 2*D* model completely independent of the *z*-direction coordinates. The ideal dipole field employed in this study is given by [23]

(4) B=−Bmax⁡2(x2+y2)5/2[2x2−y23xy0].

## 4. Computational Details

The simulations are performed using the commercial CFD code, ANSYS CFX. The computational domain consists of a cylinder with diameter, *D*_*c*_, enclosed by a second cylinder with a diameter of 40*D*_*c*_. The domain extends along the cylindrical axis for a length of 2*D*_*c*_. The mesh contains 201,000 elements uniformly biased towards the center cylinder. The entrance region, that is, the inlet is specified as an “inlet” boundary condition, the outflow region a “supersonic outlet,” and the two sides of the domain in the *z*-direction are assigned the “symmetry” boundary condition. The wall of the cylinder itself is considered to be “adiabatic” and “nonconducting.”

As mentioned above steady state was assumed. For viscosity and thermal conductivity, a Sutherland's model was used. Electrical conductivity was derived based on the Chapman-Enskog model and is dependent on pressure and temperature, details of which are provided below.

Under the low-Re_*m*_ assumption defined by

(5) $$\begin{array}{c} \text{R}{\text{e}}_{m}=\sigma \mu_{o}UL\approx 0.001, \end{array}$$

we can neglect any induced magnetic fields and treat the magnetic field as constant [31]. This allows us to also simplify our set of equations and condense the MHD effects into a source term in the momentum and energy equations, which are the Lorentz force and Joule heat terms, respectively.

### 4.1. Electrical Conductivity

For electrical conductivity, the Chapman-Enskog method [32] was implemented. This method of calculating electrical conductivity is given by

(6) $$\begin{array}{c} \sigma =\frac{3}{4}\frac{n_{e}e^{2}}{m_{e}}\frac{1}{\sqrt{8kT/\pi m_{e}}}\frac{1}{nQ}, \end{array}$$

where *Q*, the momentum transfer cross-section of collision, is

(7) $$\begin{array}{c} Q=4.398\times 10^{-10}\frac{\;\ln\left(\Lambda \right)}{T^{2}}, \end{array}$$

Λ is a parameter equal to the ratio of the Debye shielding distance to the impact parameter for 90-degree scattering by an ion and is given by

(8) $$\begin{array}{c} \Lambda =3{\left(\frac{1}{\varepsilon_{0}}\frac{n_{s}e^{2}}{kT}\right)}^{2}\left(\frac{4\pi \varepsilon_{0}}{e^{2}}\right)kT, \end{array}$$

and the number density of air, *n*, is

(9) $$\begin{array}{c} n=\frac{N_{A}}{M_{\text{air}}}\rho , \end{array}$$

which after employing the ideal gas law can be expressed as

(10) $$\begin{array}{c} n=\frac{N_{A}}{M_{\text{air}}}\frac{P}{R_{\text{air}}T}. \end{array}$$

**Figure 1:**
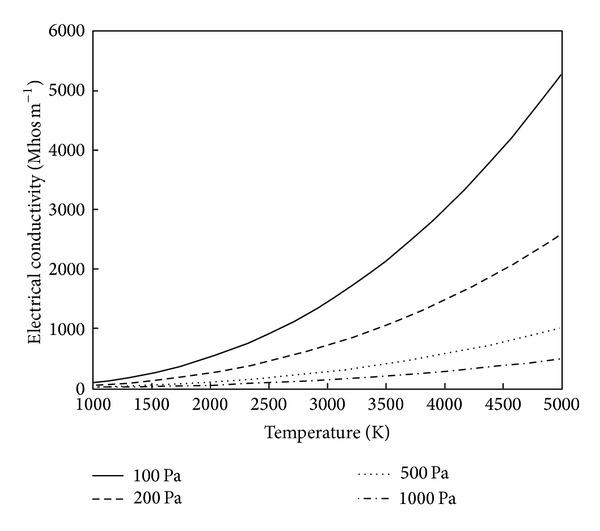
Electrical conductivity versus temperature for various pressures.

More details can be found in [32]. In these equations, *m*_*e*_ is the electron mass (=9.10938 × 10^−31^ kg), *e* is the electric charge (=1.6022 × 10^−19^ Coulombs), *k* is Boltzmann's constant (=1.38065 × 10^−23^ J/K), *ε*_0_ is the permeability of free space (=1.25664 × 10^−6^ m·kg/s^2^ A^2^), *N*_*A*_ is the Avogadro constant (=6.02214 × 10^23^ mol^−1^), *M*_air_ is the molar mass of air (=.02897 kg/mol), *R*_air_ is the specific gas constant for air (=287.04 J/kg K), and *n*_*e*_ is the electron number density. We assume a constant electron density value of *n*_*e*_ = 10^7^ cm^−3^ from electron beam ionization throughout the flowfield, which corresponds to weakly ionized airflow [33, 34]. The resulting model, which is the solution of (6)–(10), is dependent on only temperature and pressure.  Figure 1 demonstrates the increase in electrical conductivity that comes with both an increase in temperature and a decrease in pressure.

## 5. Grid Independence

**Figure 2:**
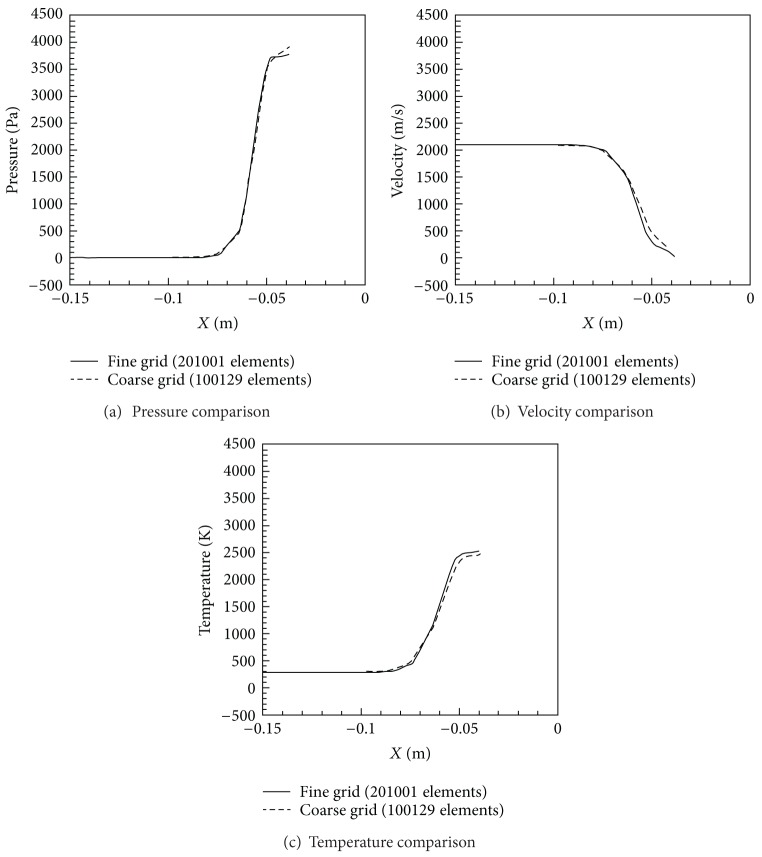
Grid independence comparison.

To ensure that the results are grid independent, calculations from the fine mesh of 201,000 elements were compared with those from a second, coarse mesh with 100,000 elements (see Figure 2). These were carried out in the absence of any magnetic field. In comparing the drag coefficients for the two meshes, a resulting error of 0.24% was obtained between the two meshes.

## 6. Results

In this section, results from simulations of hypersonic flow over a cylinder are presented. Parametric studies include two different kinds of orientations, *X*- and *Y*-oriented dipoles, and four different magnetic field strengths, with *B*_max⁡_ = 0 T, 0.2 T, 1.0 T, and 2.0 T. Analysis includes comparisons of velocity, temperature, Lorentz force, and Joule heat.

### 6.1. *X*-Oriented Magnetic Field

Equation (4) gives the magnetic field dipole oriented in the *X*-direction. The negative sign in front of *B*_max⁡_ implies that the flux vectors are oriented in the negative *X*-direction.

**Figure 3:**
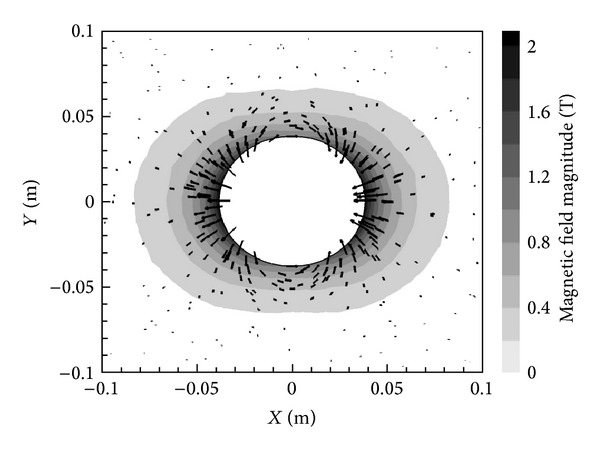
Magnetic field vectors and contours of the magnitude for an *X*-oriented dipole with *B*_max⁡_ = 2 T.

Figure 3 presents the magnetic field vectors according to the *X*-oriented dipole and also contours showing the magnitude of the magnetic field itself, calculated as $|B|=\sqrt{B_{x}^{2}+B_{y}^{2}}$.

**Figure 4:**
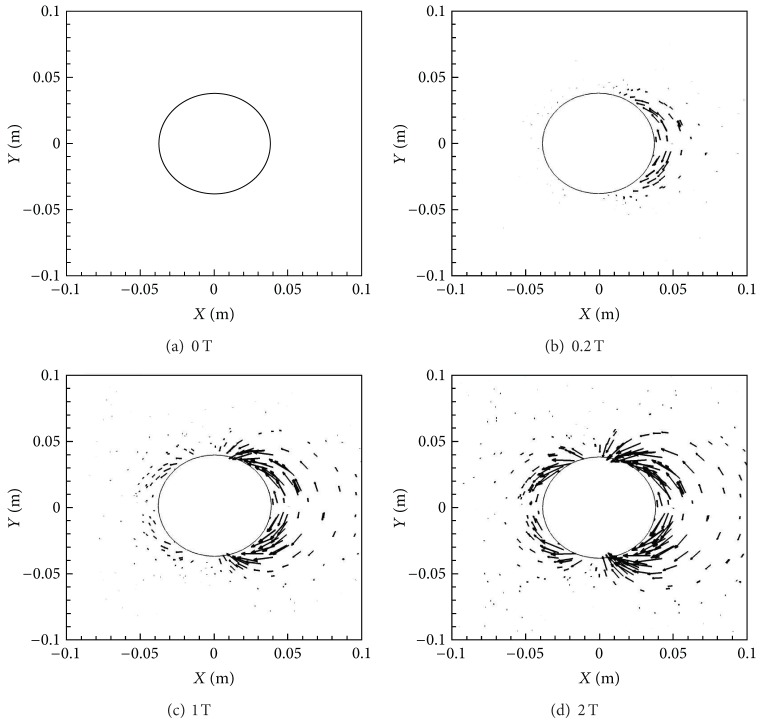
Lorentz force vectors for various *B*_max⁡_ with *X*-orientation.

Figure 4 shows the Lorentz force vectors for the four different magnetic field cases, that is, *B*_max⁡_ = 0, 0.2, 1.0, and 2.0 T. From these figures it is evident that the Lorentz forces are larger behind the cylinder compared to the front in all the cases, with the effects in the front subsequently increasing with *B*_max⁡_. The Lorentz force is directly proportional to magnetic field, *B*, current density, *J*, and electrical conductivity, *σ*. While *B* is symmetric across the front and back, *σ* is considerably larger behind the cylinder compared to the front as a result of higher temperatures and lower pressures. This in turn causes the *J* to be bigger in these regions as well, making the Lorentz force effects more significant behind the cylinder.

**Figure 5:**
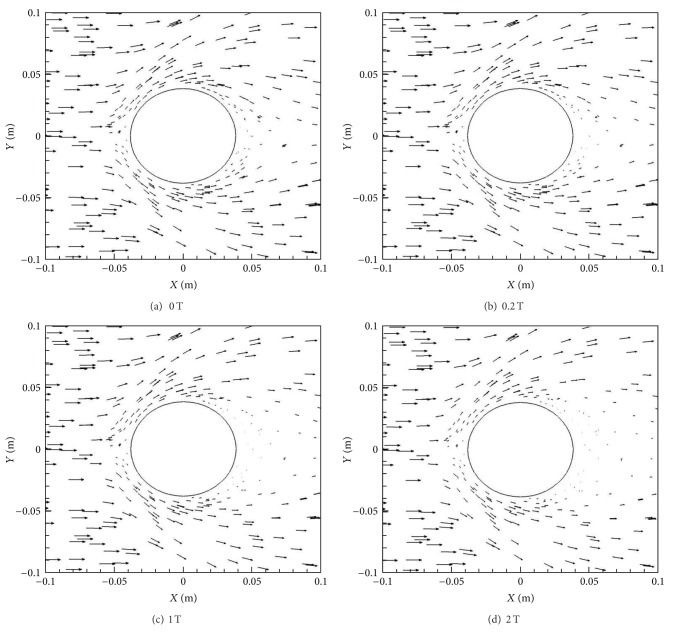
Velocity vectors for various *B*_max⁡_ with *X*-orientation.

Figure 5 presents the velocity vectors for the same four cases. It can be observed that, with an increase in magnetic field from 0 T to 2.0 T, the velocity is considerably slowed down, especially behind the cylinder due to the damping effect of the Lorentz force. Since the Lorentz forces are larger behind the cylinder due to the reasons mentioned above, velocity is influenced much more behind the cylinder compared to the front.

**Figure 6:**
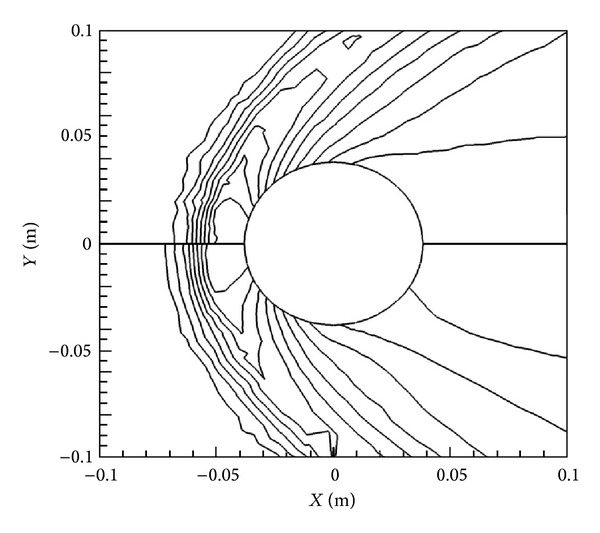
Comparison of density contours without magnetic field (top) and with *B*_max⁡_ = 2 T oriented in *X*-direction (bottom).

**Figure 7:**
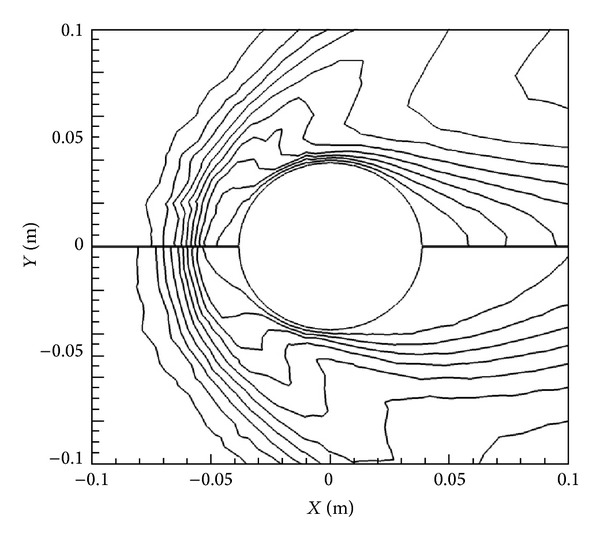
Comparison of  *U* velocity contours without magnetic field (top) and with *B*_max⁡_ = 2 T oriented in *X*-direction (bottom).

The effect that the Lorentz force has on the flow is clearly demonstrated in Figures 6 and 7, where density and velocity contours are shown for the lowest and highest magnetic field cases, that is, *B*_max⁡_ = 0 T and *B*_max⁡_ = 2.0 T on top and bottom. There is a marked reduction in velocity immediately around the cylinder, especially behind it, for the 2.0 T case, as the flowlines are pushed out further from the cylinder. The same kind of effect is also noticeable in the density contours in Figure 6 as the shock location moves away from the cylinder towards the front with the addition of the magnetic field.

**Figure 8:**
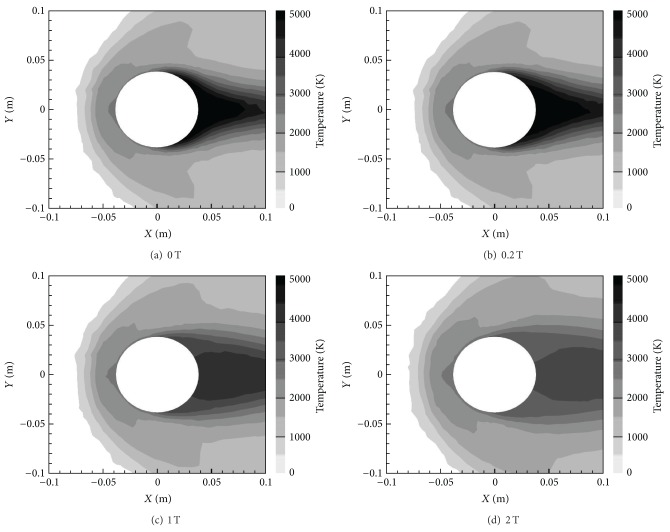
Temperature contours for various *B*_max⁡_ with *X*-orientation.

Figure 8 shows the temperature contours of the four magnetic field cases of the *X*-oriented dipole. These contours also show a change, as with increasing *B*_max⁡_ comes a reduction in overall wall-adjacent temperature around the cylinder. More specifically, the temperature around the cylinder tends towards more uniformity with an increasing magnetic field. This is because the Lorentz force that dampens the flow also reduces the convection heat transfer effects, thereby making conduction more dominant and temperature more uniform [11, 14].

**Figure 9:**
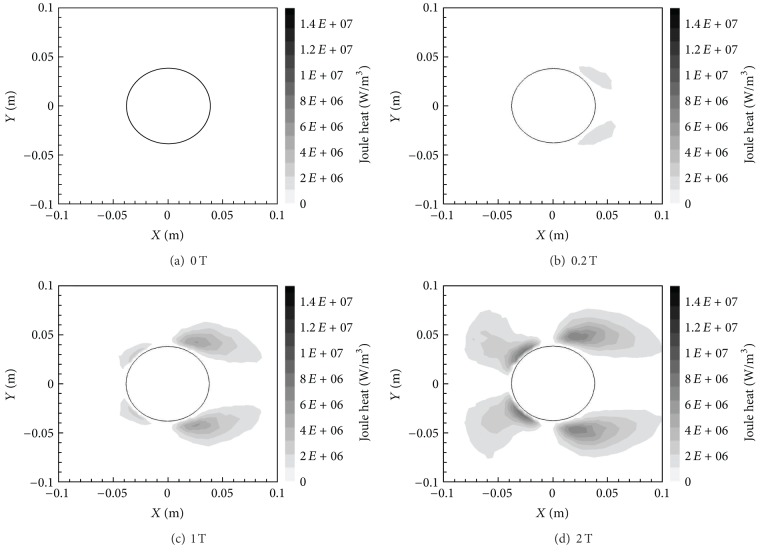
Joule heat contours for various *B*_max⁡_ with *X*-orientation.

At the same time, however, Joule heat increases with *B*_max⁡_ as shown in Figure 9. This figure presents the Joule heat contours in the entire domain for the different cases. Joule heat is calculated as *J* ^2^/*σ*, where *J* is the current density magnitude. Subsequently, in Figure 9 the Joule heat is considerably higher in those areas with both a higher current density, *J*, and a relatively lower electrical conductivity, *σ*. The electrical conductivity is the highest right behind the cylinder and right in front of the cylinder due to higher temperatures and/or lower pressures. Every other region around the cylinder has a lower electrical conductivity. Furthermore, since the magnetic field is *X*-oriented, the current density is the highest in an *X*-oriented elliptical region around the cylinder. These two factors determine the distribution of Joule heat pattern shown in Figure 9. Although Joule heat does lead to an increase in the temperature of the flow, it is overcome by the reduction in temperature around the cylinder due to Lorentz force effects.

### 6.2. *Y*-Oriented Magnetic Field

**Figure 10:**
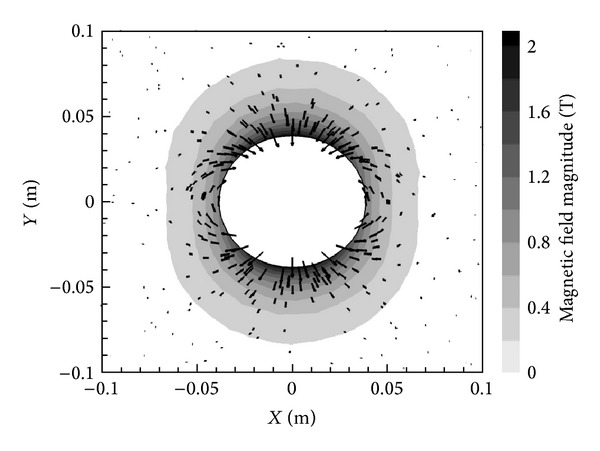
Magnetic field vectors and contours of the magnitude for a *Y*-oriented dipole with *B*_max⁡_ = 2 T.

This section presents results from MHD simulations of electrically conducting flow around a cylinder, where the magnetic field of different strengths is generated using a *Y*-oriented dipole, with the rest of the boundary conditions being exactly the same as in the previous section.  Figure 10 shows the contours of magnetic field strength along with the magnetic field vectors. Again, due to the direction of magnetic flux, the magnetic field vectors are oriented in the negative *y*-direction.

Figure 14 shows the Lorentz force vectors for the four *Y*-oriented magnetic dipole cases of *B*_max⁡_ = 0 T, 0.2 T, 1 T, and 2 T. The Lorentz force is primarily oriented along the top and bottom of the cylinder. This is expected since the magnetic field has its maximum strength in these regions. This change in location and direction of the Lorentz force predicates practically all the flow changes between the two magnetic dipole orientations.

**Figure 11:**
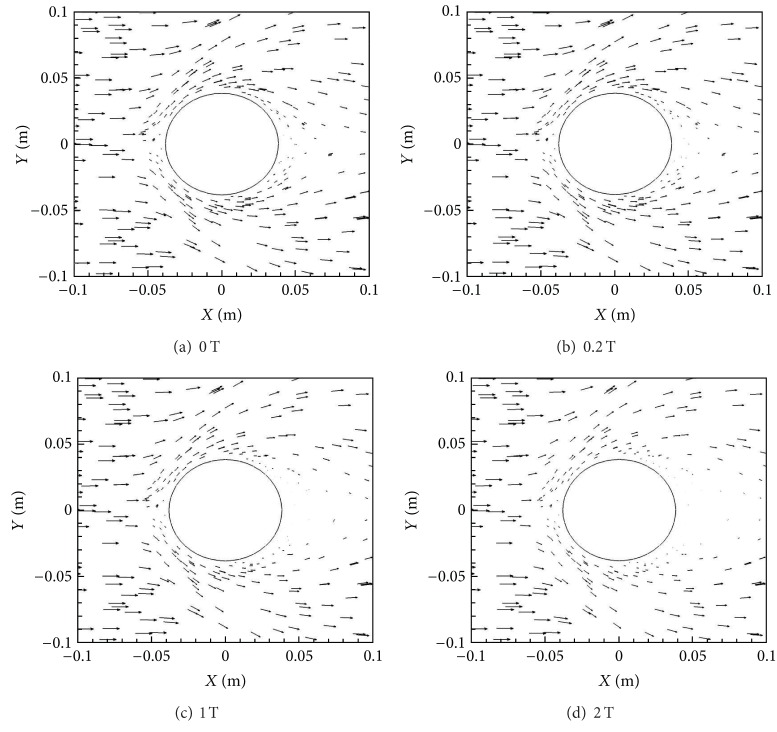
Velocity vectors for various *B*_max⁡_ with *Y*-orientation.

Figure 11 displays the vectors of velocity for the four cases. Here, too, the addition of the magnetic field causes a considerable change in flow patterns, which start to become increasingly obvious at the highest magnetic field case. In addition, the flow in the *Y*-oriented dipole cases is directed more in the transverse direction compared to the *X*-oriented dipole case, and this becomes only very obvious in the *B*_max⁡_ = 2 T case. This is primarily due to the Lorentz force effects being dominant on the top and bottom of the cylinder.

**Figure 12:**
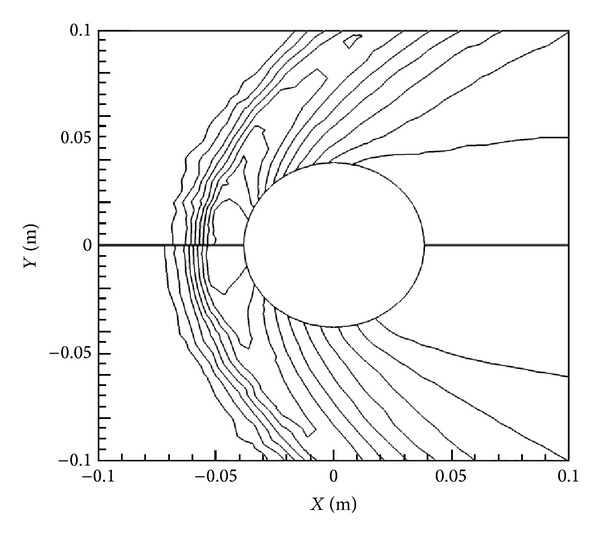
Comparison of density contours without magnetic field (top) and with *B*_max⁡_ = 2 T oriented in *Y*-direction (bottom).

**Figure 13:**
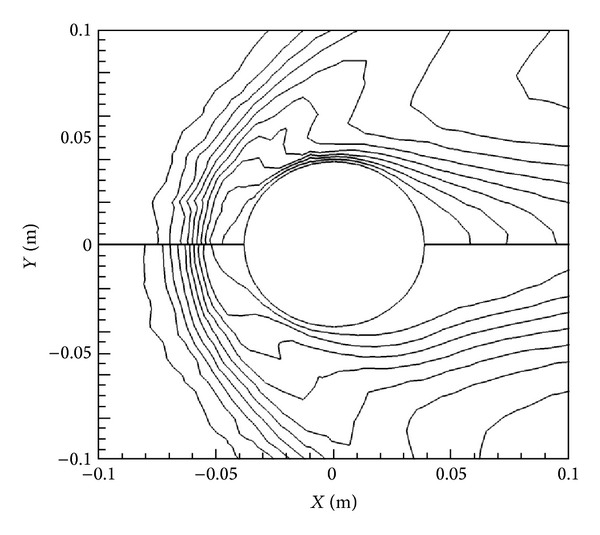
Comparison of *U* velocity contours without magnetic field (top) and with *B*_max⁡_ = 2 T oriented in *Y*-direction (bottom).

**Figure 14:**
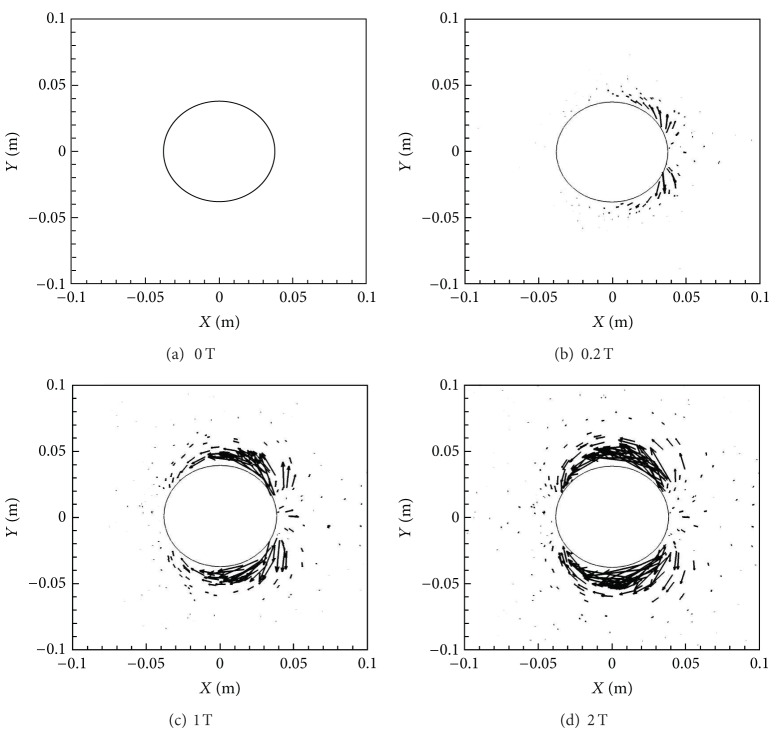
Lorentz force vectors for various *B*_max⁡_ with *Y*-orientation.

Figures 12 and 13 directly compare the velocity and density contours for the two cases *B*_max⁡_ = 0 T and 2 T. Here, in the same manner as with the *X*-oriented case, it is possible to directly see the influence of the *Y*-oriented magnetic field. Just as with the *X*-orientation, the influence of the magnetic field is starkly visible, as the contours of velocity and density are pushed out and away from the cylinder. Note, however, the increased effect in the *Y*-direction and slight decrease in effect in the *X*-direction, in comparison to the *X*-oriented dipole cases. It is obvious that the velocity contours are being pushed out now in the transverse direction to the flow. This is due to the direction of the Lorentz force, which has now changed with the differing orientation of the magnetic field.

**Figure 15:**
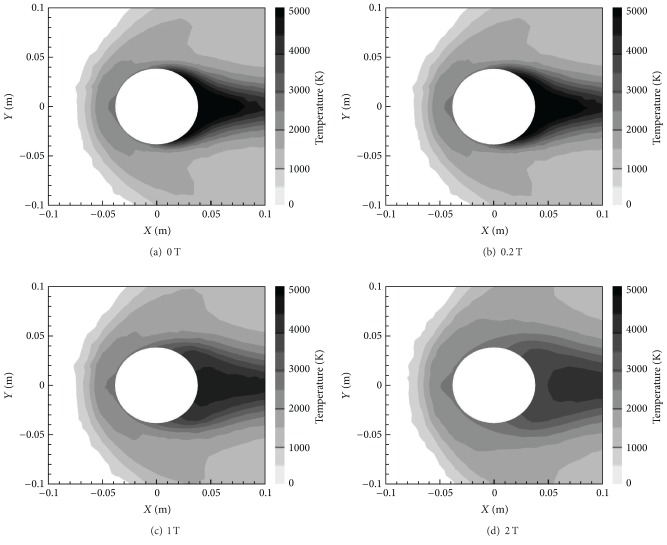
Temperature contours for various *B*_max⁡_ with *Y*-orientation.

**Figure 16:**
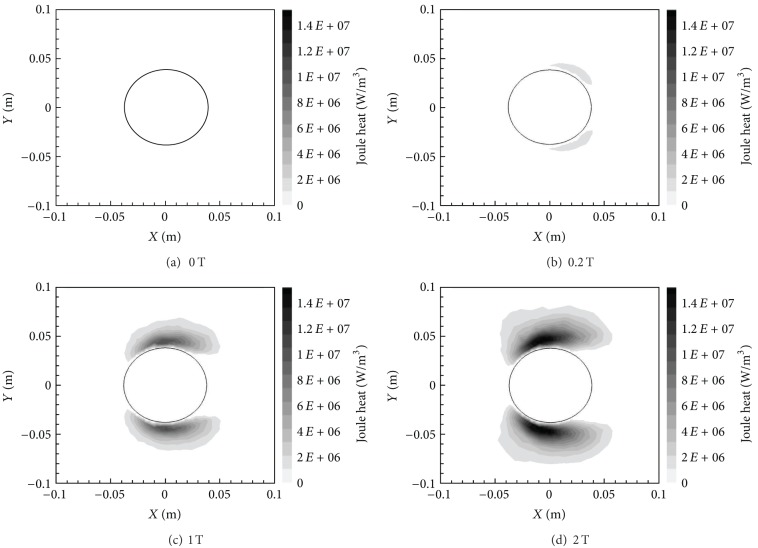
Joule heat contours for various *B*_max⁡_ with *Y*-orientation.

Figure 15 shows the temperature contours for the various *B*_max⁡_ values. Similar to the *X*-oriented field, there is a demonstrable decrease in wall-adjacent temperature around the cylinder. However, this decrease is not quite as large as the drop in values manifested by the *X*-oriented field. A quantitative analysis of the reduction in temperature is provided in Table 1. The reduction in temperature is again due to the damping of flow velocities by the Lorentz force (see discussion above for the *X*-oriented field case).  Figure 16 reveals the contours of Joule Heat for the *Y*-oriented magnetic dipole fields. Joule heat is now very low everywhere except along the top and bottom regions around the cylinder, this time, due to the *Y*-oriented magnetic field. There is an increase in the maximum Joule heat values compared to the *X*-oriented magnetic field, which is mainly because the region where electrical conductivity is the lowest (top and bottom) coincides with the region where the magnetic field or current density is the highest.

**Figure 17:**
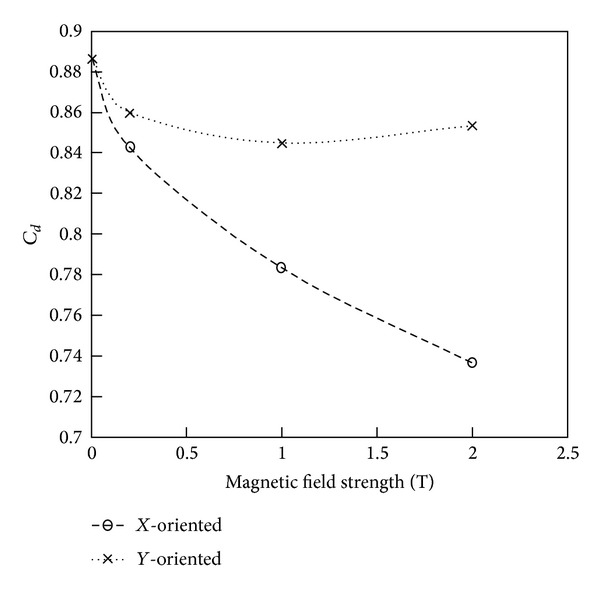
Comparison of drag coefficient for both *X*- and *Y*-oriented magnetic fields.

Lastly, we compare the effect that the magnetic field magnitudes and orientations have on the resulting drag coefficients, average wall-adjacent temperatures, and shock distance for all the cases.  Figure 17 compares the drag coefficient and Table 1 illustrates quantitatively the effect of magnetic field and orientation on drag coefficient, average wall temperature, and shock-standoff distance. The percentage reductions in variables are calculated as

(11) %  change  =  Zero  magnetic  field  value−Finite  magnetic  field  value    Zero  magnetic  field  value  .

From Figure 17, it is clear that the drag coefficient reduces for both orientations with increasing magnetic field. It is, however, observed that, for the *Y*-oriented field, *C*_*d*_ starts to increase for the highest magnetic field of 2 T. From Table 1 it can be seen that with an increase in magnetic field, both drag coefficient and the average wall-adjacent temperatures are progressively reduced and the shock-standoff distance is increased compared to the no-magnetic field case, although the percentage change in values is higher for the *X*-oriented dipole, especially for the drag coefficient where the *X*-oriented field results in four times the drag reduction. For the *Y*-oriented field, *C*_*d*_ starts to increase at higher magnetic fields because of an increase in pressure drag. This increase is mostly due to the significant reduction in flow velocities on the top and bottom of the cylinder (with the *Y*-oriented field being the strongest on the top and bottom), which eventually leads to increased pressures in these regions. The change in shock-standoff distance presented here is calculated as the distance between the discontinuities in density from the cases with and without magnetic field and it is observed that there is up to a 5.4% change in this distance with the *X*-oriented field.

Overall, it was observed that the *X*-oriented field resulted in stronger effects compared to the *Y*-oriented field at the same magnetic field strength, that is, effects in terms of reduction in flow velocities, overall temperatures, average wall temperatures, and drag coefficients. This is because the *X*-oriented field obviously has its strongest magnetic field strength in a direction aligned with the primary flow direction, which in turn means that the Lorentz force is also aligned with this direction. Hence, the Lorentz force has its maximum influence for the *X*-oriented field case since it is primarily parallel to the direction of the flow, while, for the *Y*-oriented field case, it is perpendicular.

As mentioned in the beginning of this paper, there have been quite a few studies in this area over the last decade, and many of them demonstrated similar effects as the ones observed here. Particularly relevant to the current study is the work by Bisek et al. [23], who carried out a computational investigation of near-hypersonic flow of argon gas over a hemispherical body with a dipole magnetic field and noticed similar effects on shock-standoff distance. Utilizing several electrical conductivity models, they achieved up to approximately 16% increase in shock-standoff distance with *B*_max⁡_ = 0.28 T. Numerical and experimental work by Bityurin and coworkers [17, 35, 36] on flow over a cylinder also showed an increase in shock distance (up to a 45% increase with *B*_max⁡_ = 0.45 T), as well as a decrease in temperature around the cylinder with the application of a magnetic field. Gülhan et al. performed an experimental study on partially-ionized argon gas and found up to a 44% decrease in surface temperature [37]. Grigoriadis et al. [22] Shatrov and Gerbeth [27], and Zhang et al. [28] all demonstrated effects that magnetic field and Lorentz force can have on drag reduction and encountered varying degrees of effectiveness in reducing drag. Zhang et al., for instance, found the drag coefficient to decrease linearly, even into a negative drag coefficient, with increasing interaction parameter (analogous to increasing *B*_max⁡_). This decrease was dependent on both the orientation and intensity of magnetic field strength, in line with our results. The comparatively lower magnitude that some of our results display in relation to previous studies (e.g., a 5.4% change in shock-standoff distance versus 45% noticed in [36]) can be attributed to the relatively lower electrical conductivity encountered in this study.

## 7. Conclusions

 The effects of a magnetic field applied to partially ionized laminar hypersonic flow over a cylinder were considered in this paper. Boundary values and the properties of the ionized air were chosen to simulate the environment of earth's atmosphere at a height of 50,000 m. As the resulting flow was characterized by a low-magnetic Reynolds number, the MHD effects were distilled down to two source terms—the Lorentz force and Joule heat—which were added to the momentum and energy equations, respectively. These two variables drove the changes that occurred in the flow with the addition of the magnetic field. Two magnetic fields, axial- and transverse-oriented dipoles, were compared.

The results show a decrease in flow velocities, especially behind the cylinder, decrease in flow temperature near the entire cylinder surface, an increase in the shock-standoff distance, and a decrease in the drag coefficient due to the damping effects of the Lorentz force. Effects increase with *B*_max⁡_ for the *X*-oriented field, while the *Y*-oriented field displays a lower overall effect, and in particular a lower decrease in reduction of the drag coefficient. These results are similar to findings of other researchers and add to the evidence of the benefits MHD can provide in future space vehicles. Future considerations include addressing transient and turbulent flows and further advances in weakly ionized atmospheric air electrical conductivity modeling.

## Figures and Tables

**Table 1:** Percent changes in *C*_*d*_, average wall-adjacent temperature, and shock-standoff distance for various *B*_max⁡_.

Case	*C*_*d*_	Average wall-adjacent temperature	Shock distance
0.2 T *X*-oriented	−5.0%	−3.2%	0.14%
1 T *X*-oriented	−11.7%	−13.5%	1.5%
2 T *X*-oriented	−17.0%	−18.5%	5.4%
0.2 T *Y*-oriented	−3.0%	−1.1%	0.10%
1 T Y-oriented	−4.7%	−10.1%	1.2%
2 T *Y*-oriented	−3.7%	−17.5%	4.5%

## Nomenclature

*N*_*A*_: Avogadro constant
*k*: Boltzmann's constant
*L*: Characteristic length
*ρ*: Mass density
*D*: Diameter
*μ*: Dynamic viscosity
*Q*: Electron collision cross-section
*e*: Electric charge
*σ*: Electrical conductivity
*n*_*e*_: Electron number density
*m*_*e*_: Electron mass
*J*_*H*_: Joule heat
*δ*: Kronecker delta function
*F*_Lor_: Lorentz force
*η*: Magnetic diffusivity
*μ*_*o*_: Magnetic permeability of free space
*B*_max⁡_: Max value of magnetic field
*n*: Particle concentration
*ε*_0_: Permeability of free space
*p*: Pressure
*h*_tot_: Specific total enthalpy
*T*: Temperature
*λ*: Thermal conductivity
*U*: Velocity vector
*τ*: Viscous stress tensor.
